# Real-world evidence of health outcomes and medication use 24 months after bariatric surgery in the public healthcare system in Brazil: a retrospective, single-center study

**DOI:** 10.6061/clinics/2020/e1588

**Published:** 2020-04-06

**Authors:** Denis Pajecki, Flavio Kawamoto, Anna Carolina Batista Dantas, Priscila Caldeira Andrade, Nissia Capello Brasil, Silvio Mauro Junqueira, Fernanda Maria Pirozelli de Oliveira, Rodrigo Antonini Ribeiro, Marco Aurelio Santo

**Affiliations:** I Divisao de Cirurgia, Unidade de Cirurgia Bariatrica e Metabolica, Departamento de Gastroenterologia, Hospital das Clinicas (HCFMUSP), Faculdade de Medicina, Universidade de Sao Paulo, Sao Paulo, SP, BR; II Johnson & Johnson Medical Devices, Sao Paulo, SP, BR; III HTAnalyze Consulting, Porto Alegre, RS, BR

**Keywords:** Open Roux-en-Y Gastric Bypass, Healthcare Costs, Real-World Evidence, Delivery of Healthcare

## Abstract

**OBJECTIVES:**

The number of bariatric procedures has significantly increased in Brazil, especially in the public Unified Health System. The present study describes health outcomes and medication use in obese patients treated in a major hospital that performs publicly funded surgery in Brazil.

**METHODS:**

A retrospective, single center study was conducted to collect real-world evidence of health outcomes and medication use in 247 obese patients (female, 82.2%) who underwent open Roux-en-Y gastric bypass. Changes in weight and body mass index (BMI), presence of apnea, hypertension, and type 2 diabetes (T2D), and medication use (hypertension, diabetes, and dyslipidemia) were assessed preoperatively and up to 24 months postoperatively. The mean cost of medications was calculated for the 12-month preoperative and 24-month postoperative periods.

**RESULTS:**

During the surgery, the mean age of patients was 43.42 years (standard deviation [SD], 10.9 years), and mean BMI was 46.7 kg/m^2^ (SD, 6.7 kg/m^2^). At 24 months, significant declines were noted in weight (mean, -37.6 kg), BMI (mean, -14.3 kg/m^2^); presence of T2D, hypertension, and apnea (-29.6%, -50.6%, and -20.9%, respectively); and number of patients using medications (-66.67% for diabetes, -41.86% for hypertension, and -55.26% for dyslipidemia). The mean cost of medications (total costs for all medications) decreased by >50% in 12–24 postoperative months compared to that in 12 preoperative months.

**CONCLUSION:**

Roux-en-Y gastric bypass successfully reduced weight, BMI, and comorbidities and medication use and cost at 24 months in Brazilian patients treated in the public Unified Health System.

## INTRODUCTION

There is consistent evidence that surgical treatment of obesity results in more favorable weight loss and weight-associated comorbidity outcomes compared with nonsurgical interventions 1,2, with a positive impact on preexisting diabetes and hypertension, among others 3,4. A lower risk of developing type 2 diabetes (T2D), hypertension, angina, myocardial infarction, and obstructive sleep apnea after bariatric surgery has also been reported 4. The improvement or resolution of comorbidities is likely to entail reduced medication use and expenditure following bariatric surgery 3.

In 2014, Brazil was second only to the United States in the number of bariatric/metabolic surgical procedures, with >95,000 surgeries in the country 5. In fact, Brazil has witnessed a steady growth in the number of bariatric procedures, especially in the public Unified Health System (SUS) 6,7. Previous studies on the Brazilian population have focused on some aspects of medication use and cost, including assessments before and after bariatric surgery 3 and impact of surgery on metabolic syndrome components and associated medications 8. However, these studies reported extremely short-term results only, at 6 and 2 postoperative months, respectively. A historical cohort on patients with private health insurance who were analyzed 1 year before and 1 year after bariatric surgery to determine cost and use of healthcare services 9 did not include information on medication use, since medications are typically paid out of pocket by these patients in Brazil. Other studies with follow-up ranging from 12 months to 10 years 10-13 have provided only limited information on medication use and no information on medication cost or included cost of medications only as part of a composite variable.

Therefore, this study aimed to describe health outcomes and medication use from waitlisting for bariatric surgery until 24 months postoperatively in obese patients treated in a major hospital providing public healthcare in Brazil.

## MATERIALS AND METHODS

We performed a retrospective, single-center study to collect real-world evidence of health outcomes and medication use following gastric bypass. Medical records were reviewed to identify eligible patients, and data covering the period from wait listing for bariatric surgery (baseline) up to 24 months postoperatively were collected. The following variables were assessed preoperatively and at 6, 12, and 24 months postoperatively: weight and body mass index (BMI), presence of comorbidities (apnea, hypertension, T2D), and medication use (hypertension, T2D, and dyslipidemia). The mean cost of medications was calculated for the period of 12 months preoperatively and 0–12 and 12–24 months postoperatively. The mean cost of medications used during the entire 24-month follow-up was also calculated. The price of medications was obtained from the Ministry of Health (http://portalms.saude.gov.br/gestao-do-sus/economia-da-saude/banco-de-precos-em-saude).

### Setting and participants

The study was performed at Hospital das Clínicas de São Paulo, a university hospital providing publicly funded care. Adult patients undergoing SUS-reimbursed open Roux-en-Y gastric bypass from January 2010 to July 2012 were eligible for the study. The inclusion criteria were availability of 24-month postprocedure follow-up data, age ≥18 to ≤65 years, and BMI ≥40 kg/m^2^ (not responding to conservative treatment for at least 2 years or having a life-threatening condition resulting from comorbidities) or BMI ≥35 mg/m^2^ in the presence of at least one severe comorbidity associated with obesity (hypertension, diabetes, etc.) 14,15, as determined by the Brazilian Public Health Guidelines at the time the study was conducted.

The exclusion criteria were pregnancy, Roux-en-Y gastric bypass performed for revision or with other surgical procedures (multiple surgeries) except for cholecystectomy (e.g., colectomy or procedures not related to the bypass), other types of bariatric surgery (gastric banding, sleeve gastrectomy, etc.), and initial indication for other bariatric procedures subsequently converted to Roux-en-Y gastric bypass. The presence of the following conditions was also an exclusion criterion: advanced cancer, HIV, viral hepatitis, drug abuse, car accident, participation in interventional clinical trials affecting treatment standards and use of resources during the study period, and loss to postoperative follow-up.

### Statistical analysis

For numerical variables, the Shapiro-Wilk normality test was first applied to test if variables followed a normal distribution. Data that followed a normal distribution were presented as means and standard deviation. Other data were presented as median and interquartile interval. Categorical variables were expressed as number and percentage (excluding missing data).

To conduce the analyses, data cleaning was previously required. In this process, missing data were identified and excluded from the analysis. For weight measurement, missing data were identified in 1 observation during surgery, 32 at 6 months, 21 at 12 months, and 19 at 24 months (representing 32 missing observations during surgery *vs*. 6 months, 21 during surgery *vs*. 12 months, and 19 during surgery *vs*. 24 months). In the variables that indicate the presence of comorbidities, 18 missing observations were found for sleep apnea (9 missing observations at 6 months, 6 at 12 months, and 3 at 24 months postoperatively). For T2D and hypertension, 19 missing observations were identified (9 missing observations at 6 months, 7 at 12 months, and 3 at 24 months, postoperatively). For medication use and cost, no missing variables were found.

All analyses conducted in this study aimed to evaluate the variation in BMI, weight, cost, medication use, and comorbidities between the baseline and postoperative periods (6, 12, and 24 months). After the analysis of the results throughout the follow-up period, a post hoc analysis was conducted to test the differences during surgery and each of the three follow-up periods (6, 12, and 24 months postoperatively).

To evaluate the variation in weight and BMI during the follow-up, the Shapiro-Wilk normality test was used, and nonparametric tests were selected given the non-normality of data. The Friedman test was initially conducted to identify if weight differed across the periods (during surgery and after 6, 12, and 24 months), and post-hoc pairwise test was applied to identify if variables during surgery and in each of the follow-up periods were statistically significantly different.

To determine the presence of comorbidities in each period (during surgery and after 6, 12, and 24 months), a contingency table was created for three common diseases associated with BMI: T2D, hypertension, and sleep apnea.

To evaluate the difference in the use of medication pre- and postoperatively, a contingency table was made, taking into account the possible changes in intake doses of each patient. Subsequently, the Cochran's Q test was utilized to determine if the differences were statistically significant. The post hoc McNemar pairwise test was then applied to determine which two-period comparison had different medication proportions.

In the cost analysis, the price of each medication was selected from the Health Prices Database (Banco de Preços em Saúde), a system created by the Brazilian Ministry of Health to record information on public and private purchases of medications and health products. The cost of each medication for every patient was then calculated, considering that the same patient could take different doses of the same medication. After calculating the cost of each medication in the database (29 drugs), the cost for each patient was then derived as the sum of every medication. The mean cost for each period was obtained using total patients and costs. Then, the Friedman test was conducted to determine if the mean cost of medication was statistically significantly different between periods, and the post-hoc pairwise Wilcoxon test was conducted to identify if there are significant differences between surgery and each follow-up period.

## RESULTS

Of 259 potentially eligible patients, 247 fulfilled the inclusion and exclusion criteria and were finally included. The reasons for exclusions were <24 months of follow-up data (N=7) and age outside the predetermined range (N=5). The sample included 203 women (82.18%).

The mean waiting time from baseline to surgery was 1,224 days (SD, ±297.77). The characteristics of the sample at baseline and during surgery are presented in Table 1.

**Figure 1:**
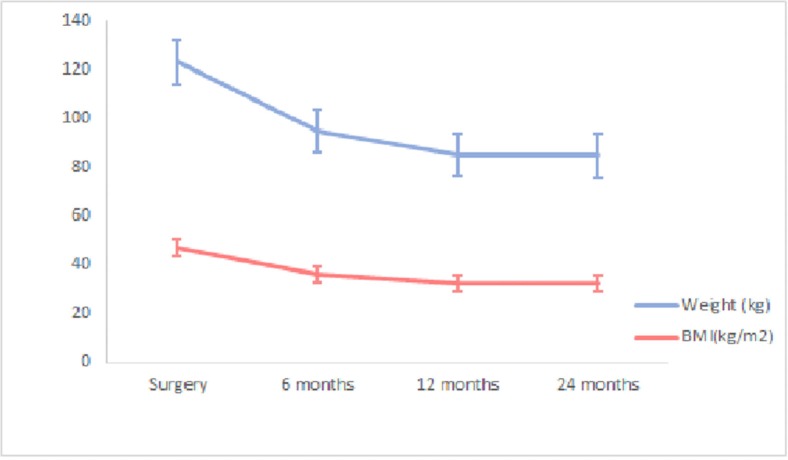
Median weight loss and BMI decline at 24 months in patients undergoing gastric bypass within the public healthcare system, Hospital das Clínicas de São Paulo, Brazil, in 2010–2012. *p*<0.001 (Friedman test). During surgery *vs*. after 6, 12, and 24 months; *p*<0.001 (Wilcoxon test). Values for weight (kg): 122 during surgery; 93 after 6 months; 83.50 after 12 months; 83 after 24 months. Values for BMI (kg/m^2^): 46.22 during surgery; 35.27 after 6 months; 31.90 after 12 months; 31.55 after 24 months.

A median weight loss of 6 kg was found at baseline and during surgery, with a median BMI decline of 2.205 kg/m^2^. Weight and BMI decreased steadily throughout the study (Figure 1). At 24 months, a median decrease in weight and BMI of 38 kg and 14.86 kg/m^2^, respectively, was observed (31.45% in relation to that during surgery, for both variables). The decrease in weight and BMI was more pronounced 12 months postoperatively.

### Comorbidities and medication use

**Figure 2:**
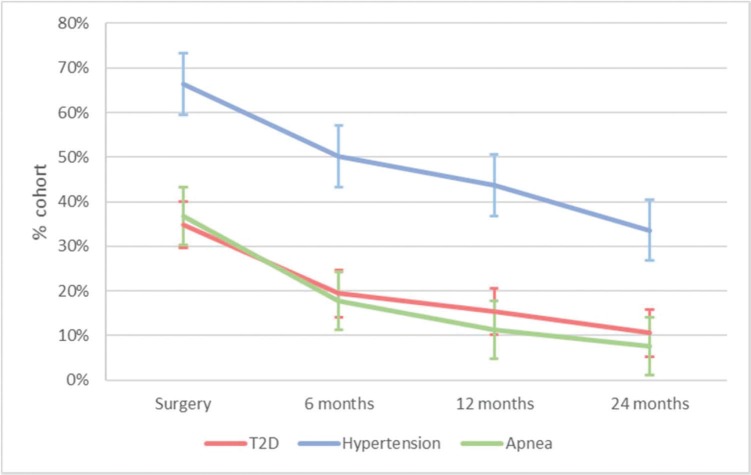
Presence of comorbidities during gastric bypass and 6, 12, and 24 months postoperatively in patients treated in the public healthcare system, Hospital das Clínicas de São Paulo, Brazil, in 2010–2012. *p*<0.001 (McNemar) during surgery *vs*. after 6, 12, and 24 months. SAH, systemic arterial hypertension; T2D, type 2 diabetes. Values for T2D: 34.82% during surgery; 19.43% after 6 months; 15.38% after 12 months; 10.53% after 24 months. Values for hypertension: 66.40% during surgery; 50.20% after 6 months; 43.72% after 12 months; 33.60% after 24 months. Values for apnea: 36.84% during surgery; 17.81% after 6 months; 11.33% after 12 months; 7.69% after 24 months.

During surgery, in 247 patients, T2D was present in 88 (35.6%), hypertension in 164 (66.4%), and apnea in 91 (36.8%). A marked decrease in the presence of comorbidities was observed at 24 months (Figure 2), with relative reduction rates at 24 months *vs*. during surgery of 29.5% for T2D, 50.6% for hypertension, and 20.9% for apnea.

The number of patients using medications to treat T2D, hypertension, and dyslipidemia also significantly decreased in the study period (Table 2): a 66.7% decrease was noted in the number of patients using medications for T2D at 24 months *vs*. baseline; the decrease was 41.9% for those for hypertension and 55.3% for those for dyslipidemia. The mean cost of medications (considering the total costs for all medications used to treat T2D, hypertension, and dyslipidemia) decreased by >50% from 12 preoperative months until 12–24 postoperative months (Table 3).

## DISCUSSION

The present study analyzed the 24-month results of 247 patients undergoing bariatric surgery in the public healthcare system in Brazil, showing a steady decline in weight, presence of comorbidities, and number of patients using medications in the observation period. As previously shown 16, the number of studies reporting long-term results of bariatric surgery, regardless of the technique used, is still limited, with most reporting follow-ups of <2 years. In that sense, the present results provide valuable real-world evidence of the maintenance of favorable outcomes 24 months postoperatively in a public healthcare system. This is especially important given the difficulty in consistently following patients outside structured trials 11. Our findings are also consistent with those reported by a recent study describing the long-term (7 years) weight change and health status following Roux-en-Y gastric bypass and laparoscopic adjustable gastric banding 17, which show sustained weight loss and comorbidity remission specifically with bypass procedures.

Because of the well-known relationship between obesity and several debilitating health conditions 2, such as diabetes, whose control is still largely pharmacological, we also investigated the use and cost of medications before and after bariatric surgery. Although a dramatic decrease in the number of patients using medications was noted (66.7%, 41.9%, and 55.3% for T2D, hypertension, and dyslipidemia medications, respectively), the straightforward savings calculated in Brazilian currency were insignificant in contrast to the cost of surgery. This might be related to the fact that these surgeries were performed in the public healthcare system in Brazil, in which the cost of prescription medications is unusually low, since newer drugs, such as medications from the DPP-4, GLP-1, and SGLT-2 classes, and newer insulins are not reimbursed by the public healthcare system. However, for individuals who rely on private healthcare insurance (does not cover medication expenses), the decrease in the need for medications might translate into real savings. This might also be the case in other countries or healthcare systems where newer and more expensive drugs are employed. It is noteworthy that Kelles et al. 9 reported a mean cost of US$ 3,227.16 for bariatric surgery in the private healthcare system in Brazil *vs*. US$ 1,380.74 for the same open surgery in the SUS.

Moreover, as reported by Larsen et al. 18, results on the ability of bariatric surgery to generate healthcare savings are inconsistent. In their study, the authors investigated the impact of bariatric surgery on healthcare costs, social transfer payments, and income in a surgical *vs*. nonsurgical group in 7 years (3 years preoperatively, year of surgery, and 3 years postoperative) in Denmark. The authors concluded that none of these aspects were affected by bariatric surgery. However, the cost of medications was significantly lower in the surgical group, especially that of antidiabetic medications. Another study from Denmark 19 found large reductions in treatment of metabolic syndrome-related conditions and use of inhalants for obstructive airway diseases and glucocorticoid 3 years after gastric bypass, with increase in the use of neuropsychiatric drugs. Regardless of the increased use of some drugs, these findings underscore the positive clinical impact of bariatric surgery, which might be difficult to translate into measurements of direct health costs; the metabolic benefits of weight loss *per se* should not be overlooked. Additionally, specific characteristics of different countries and healthcare systems might also play a role in the cost, savings, and improvement in bariatric surgery.

An aspect that deserves to be explored further in future studies is the quality of life (QoL) following gastric bypass. In Brazil, preliminary results from the state of Goiás show improvement in QoL following gastric bypass in 50 patients followed for a minimum of 3 months (n=14) and maximum of >100 months (n=3) 13. Moreover, a systematic review 20 of prospective studies (most of which had fair to good methodological quality) showed a significant short- and long-term increase in QoL after bariatric surgery. However, the authors revealed that the questionnaires used for assessment are too general, complicating the association of QoL results with medical benefits.

The present study also included publicly treated patients facing a multiyear wait before the bariatric procedure. The recorded waiting period of approximately 3 years is typical for the SUS 21. In Canada, a recent study indicates a waiting time of >12 months between referral and specialist consultation plus an additional 6–18 months until surgery 22, which could translate into comparable wait times. Nevertheless, the health status of our sample was worse than that of patients treated by the Ontario Bariatric Network, with similar age (44.6 *vs*. 43.2 years in Ontario *v*s. São Paulo) and proportion of women (81.9% *vs*. 82.18%) – with our patients showing a fairly higher rate of obstructive sleep apnea, diabetes, and hypertension (30.9%, 29.6%, and 27.2% in Ontario *vs*. 36.8%, 35.6%, and 66.4% in São Paulo) 23. It should be noted that we detected an increase in the number of patients with T2D, hypertension, and apnea and those using medications between waitlisting and surgery.

Despite the poor health status of our sample and the deleterious effects possibly associated with long waits for surgery 24, the 24-month results we obtained were satisfactory. Among other aspects, this might reflect the experience of the surgical team in São Paulo, since the learning curve for proficiency in bariatric surgery has been especially challenging, with one study claiming a threshold of 100 cases 25 and a more recent study from the state of Rio de Janeiro (providing mixed private and public healthcare) established a cutoff as 500 cases 26.

Some limitations of the present study must be acknowledged. Given the specific patient management system used by the study center, the pre- and postoperative status of patients may not be generalizable to other populations. Furthermore, the quality of the information collected may have been compromised by the absence of control mechanisms in clinical practice, a limitation inherent to the retrospective design. Finally, the measurement of medication costs alone is not sufficient to draw conclusions regarding economic advantages of bariatric surgery in the healthcare system.

## CONCLUSIONS

The present 24-month follow-up of patients treated in the public healthcare system in Brazil shows that gastric bypass is an advantageous procedure that results in significant weight loss. The presence of diabetes, hypertension, and apnea decreased in association with gastric bypass, as did medication use. A significant decrease was also observed for the cost associated with medications. Further studies should focus on longer follow-ups and comprehensive social and economic assessments of the contribution of gastric bypass to the healthcare system.

### Conflicts of Interest

This study was funded by Johnson & Johnson do Brasil Ind. e Com. de Produtos para Saúde Ltda. The authors DP, MAS, FK, and ACBD report grants from Johnson & Johnson Medical Devices, Brazil during the conduct of the study. The authors NCB and FMPO report personal fees from Johnson & Johnson Medical Devices, Brazil during the conduct of the study.

## AUTHOR CONTRIBUTIONS

Pajecki D was responsible for the study conception, data curation, formal analysis, funding acquisition, investigation, methodology, project administration, providing software resources, supervision, validation, visualization, manuscript drafting, review and editing. Kawamoto F and Dantas ACB were responsible for the data curation, formal analysis, investigation, methodology. Andrade PC and Junqueira SM were responsible for the study design support, study strategy, analysis technical support. Brasil N was responsible for the conception, formal analysis, funding acquisition, investigation, methodology, project administration, manuscript original drafting, review and editing. Oliveira FMP was responsible for the formal analysis, manuscript drafting, review and editing. Ribeiro RA is the consultant for performing analysis and manuscript writing. Santo MA was responsible for the data curation, formal analysis, investigation, methodology, manuscript drafting, review and editing.

## Figures and Tables

**Table 1:** Characteristics of patients undergoing gastric bypass in the public healthcare system, Hospital das Clínicas de São Paulo, Brazil, in 2010–2012.

Variable	Baseline	Surgery
Age in years, mean (SD)	41.31 (10.9)	43.42 (10.9)
Female sex, n (%)	203 (82.2)	203 (82.2)
Body mass index (kg/m^2^), median (IQR)	48.41 (7, 56)	46.22 (7, 38.7)
Presence of comorbidities, n (%)		
Type 2 diabetes	81 (32.8)	88 (35.6)
Hypertension	160 (64.8)	164 (66.4)
Apnea	84 (34.0)	91 (36.8)
Medication use, n (%)		
Type 2 diabetes	59 (23.9)	60 (24.3)
Hypertension	132 (53.4)	141 (57.9)
Dyslipidemia	33 (13.4)	38 (15.4)

**Table 2:** Use of medications before and after gastric bypass in patients treated within the public healthcare system, Hospital das Clínicas de São Paulo, Brazil, in 2010–2012.

	Patients using medication for					
	Type 2 diabetes^a^^b^		Hypertension^a^^b^		Dyslipidemia^a^^c^	
Time point^d^	n (%)	% variation since baseline	n (%)	% variation since baseline	n (%)	% variation since baseline
During surgery	60 (24.3)	-	141 (57.1)	-	38 (15.4)	-
After 6 months	27 (10.9)	-55.0	161 (46.9)	-17.7	28 (11.3)	-26.3
After 12 months	22 (8.9)	-63.3	98 (39.7)	-30.5	19 (7.7)	-50.0
After 24 months	20 (8.1)	-66.7	78 (33.2)	-41.9	17 (6.9)	-55.3

a *p*<0.001 for type 2 diabetes, hypertension, and dyslipidemia (Cochran’s Q test).

b *p*<0.001 (post hoc McNemar) during surgery *vs*. after 6, 12, and 24 months.

c *p*<0.01 during surgery *vs*. after 6 months; *p*<0.001 (post hoc McNemar) during surgery *vs*. after 12 and 24 months.

d Patients using medications during surgery and 6, 12, and 24 months postoperatively.

**Table 3:** Mean cost of medications before and after gastric bypass in patients treated within the public healthcare system, Hospital das Clínicas de São Paulo, Brazil, in 2010–2012.

Mean cost of medications^a^	
Time interval^b^^c^	R$ (SD) per patient
12 months preop	151.81 (236.98)
0-12 months postop	86.53 (236.67)
12-24 months postop	66.19 (131.05)
0-24 months postop	76.36 (118.28)

a Mean cost of all medications used by patients 12 months preoperatively, 0-12 months and 12-24 months postoperatively, and for the entire postoperative period of 24 months. 1.00 USD = 3.86 BLR.

b *p*<0.001 (Friedman test).

c *p*<0.001 for 12 months preoperatively *vs*. 0-12 months, 12-24 months, and 0-24 months postoperatively (Wilcoxon test).
